# Antibody responses to equine parapoxvirus reveal a re-emerging pattern

**DOI:** 10.1186/s12917-026-05314-0

**Published:** 2026-01-24

**Authors:** Jenni Pettersson, Lev Levanov, Sanna Tervo, Katja Hautala, Kirsi Aaltonen, Mira Utriainen, Lauri Kareinen, Tuija Gadd, Tarja Sironen, Olli Vapalahti, Paula M. Kinnunen

**Affiliations:** 1 Department of Veterinary Biosciences, Faculty of Veterinary Medicine, University of Helsinki, Agnes Sjöbergin Katu 2, 00790 Helsinki, Finland; 2 Department of Virology, Faculty of Medicine, University of Helsinki, Haartmaninkatu 3, 00290 Helsinki, Finland; 3 Institute of Biomedicine, Faculty of Medicine, University of Turku and University Hospital of Turku, Kiinamyllynkatu 10, 20520 Turku, Finland; 4 Finnish Food Authority, Mustialankatu 3, 00790 Helsinki, Finland

**Keywords:** EqPPV, Parapoxviruses, Serology, Immunofluorescence assay, Western blot

## Abstract

**Background:**

Parapoxviruses (PPV) cause skin and mucous membrane signs to several animal species and humans worldwide. Equine parapoxvirus (EqPPV) was first detected in a sick horse in Finland in 2013. It is potentially zoonotic, and a similar virus has been detected in humans in the USA. In winter 2021–2022, EqPPV caused a large-scale pastern dermatitis epidemic in racehorses all over Finland. Field reports suggest that similar epidemics of unverified cause have also occurred in 2015 and 2019. The aim of this study was to develop a serological test and study the immune response, seroprevalence, and history of the virus utilizing serum samples from clinical cases and archived horse samples (2012–2022).

**Results:**

A recombinant protein-based immunofluorescent assay was established using envelope proteins B2L and F1L. EqPPV induced a fast immune response within a few days from the onset of the clinical signs. Two horses that were additionally tested a year after the disease still had similar IgG titers as a year prior. Seroprevalence peaks coincided with reported outbreaks in 2015 and 2022 (yearly variation: 1.8–14.6% [B2L] and 3.6–16.7% [F1L]).

**Conclusions:**

The results suggest that EqPPV is a re-emerging pathogen that has a potential to cause large epidemics, bringing a need for more studies and preparedness.

**Supplementary Information:**

The online version contains supplementary material available at 10.1186/s12917-026-05314-0.

## Background

Parapoxviruses (PPV) are known to cause skin and mucous membrane lesions in several animal species worldwide, most notably in domestic and wild ruminants [1]. Five virus species are officially classified: orf virus (ORFV), bovine papular stomatitis virus (BPSV), pseudocowpoxvirus (PCPV), red deer parapoxvirus (RDPV), and grey sealpox virus (GSEPV) [2]. Additionally, equine parapoxvirus (EqPPV) has recently been identified and characterized [3]. ORFV, BPSV, and PCPV are known to be zoonotic, and others are potentially zoonotic, causing painful skin lesions in humans [4, 5]. In Finland, ORFV, BPSV, PCPV, and EqPPV are detected in sheep, cattle, reindeer, horses, and, occasionally, humans.

EqPPV infection was first described in a severely sick horse in Finland in 2013 [3, 6]. Around the same time, a similar virus was identified in two humans that had been in contact with horses and donkeys in the United States [7]. The two viruses showed 99–100% nucleotide identity (100% amino acid similarity) based on a partial RNA polymerase sequence (ORF147, ORF numbers according to Delhon et al. [8]), but the short length of the sequence prevents definite conclusions on whether findings from humans in United States and horses in Finland represent the same virus species [6].

In winter 2021–2022, a pastern dermatitis epidemic occurred in racehorses in Finland. Hundreds of horses got sick with painful skin lesions in the pastern area [9]. Clinical signs lasted for a few weeks and many sick horses had to be taken out of training, leading to financial losses. PCR screening of affected horses identified EqPPV in 23/26 tested samples from different regions of the country, making it the most likely cause of the widespread epidemic. Based on a questionnaire for case and control stables, around one third of the horses in affected stables got the disease and the median time between the onset of the signs of the first and second case was three days. Interestingly, skin symptoms in humans in contact with infected horses were reported significantly more frequently by case stables than control stables [9]. Unfortunately, lack of human samples prevented the confirmation of zoonotic transmission.

The exact case number, especially before the 2021–2022 epidemic, remains unclear and the lack of serological test has prevented following the immune response of individuals and prevalence in horse population. Based on the information from the racing community and veterinarians, similar pastern dermatitis epidemics were observed in winters 2014–2015 and 2018–2019. As no dermal samples were taken until 2021, the causes of the previous epidemics have never been confirmed in the laboratory. However, knowledge about the immune response, disease history, and population immunity would be important in predicting, diagnosing, and controlling future outbreaks.

Most of the EqPPV genome has been sequenced along with a preliminary annotation [3, 9]. Based on other PPVs, envelope proteins F1L (ORF059) and B2L (ORF011) are highly immunogenic and have been used for recombinant protein-based serological assays for detecting ORFV and GSEPV antibodies [10–13]. F1L (36 kDa) is a homologue to Vaccinia virus (VACV) envelope protein H3L that is localized in intracellular mature virus (IMV) membrane and involved in virus entry into host cell [14, 15]. B2L (41 kDa) is a homologue to VACV F13L protein that is localized on the inner surface of the outer membrane and needed for formation of enveloped virus particles before they are released from the host cell [16, 17]. EqPPV F1L gene is 60–64% identical and B2L 77–79% identical to ORFV proteins in amino acid level.

The aim of this study was to establish a serological test detecting antibodies against EqPPV and study the immune response of affected horses during and after the 2021–2022 epidemic. Additionally, we aimed at acquiring information about the seroprevalence in horse population during and before the latest epidemic to understand temporal patterns and numbers of unreported infections in the disease outbreaks.

## Methods

### Serum samples

Serum samples were collected for diagnostic purposes from 26 horses from 11 stables located in southern and central Finland during the pastern dermatitis epidemic between December 2021 and March 2022. The distances between the stables varied between 10 and 270 km (approximated to the nearest 10 km; map in [9]). All the sampled horses were trotting racehorses (Additional file 1, Table S1). The herd sizes and management systems of the stables were diverse: herd size varied from small (< 10 horses) to large (> 51), and horses were kept either in individual boxes, in individual boxes with access to individual outdoor areas, housed loose, or kept in a combination of these systems (Additional file 1, Table S1). No further management data is available.

All the horses showed signs of pastern dermatitis. In twenty of the horses, EqPPV DNA had been detected by PCR, one had provided borderline results, and one had been PCR-negative [9] (Additional file 1, Table S1). Four had not been tested with PCR but the clinical signs were consistent with PCR-verified cases, and two of these horses lived at stables with PCR-verified EqPPV infection. In total, 24/26 tested horses, from which we received sera, lived with at least one PCR-confirmed stablemate.

Three horses were sampled within the first day after the clinical signs were noticed, twelve horses were sampled at some other point during the first week, and the rest were sampled at some point during the first few weeks (Additional file 1, Table S1). Additional samples were acquired from two horses (C3 and I1) a year after the disease (February 2023). Most of these horses have been previously included in an epidemiological study on the 2021–2022 outbreak [9]. Sample codes are the same between studies [9].

Three serum samples and one whole blood sample from horses sampled for diagnostic purposes before the epidemic were used as negative controls. Three of these had been tested for parapoxviruses with PCR due to showing signs of dermatitis and had turned out negative. One had earlier been tested to be positive for orthopoxvirus (OPV) IgG antibodies as part of routine diagnostics as described earlier [18]. One had been collected for diagnostics of another viral disease.

**Fig. 1:**
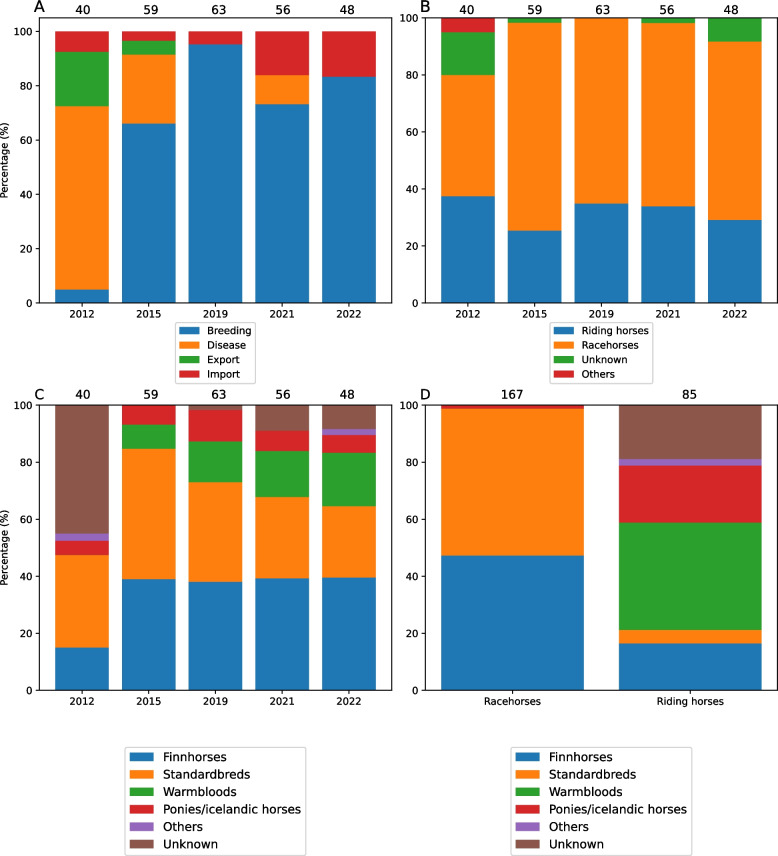
Metadata on archived samples. Distribution of reason for sampling (**A**), use (**B**), and type (**C** and **D**) grouped by collection year (**A-C**) and type (**D**). Sample numbers are shown above the pictures

A total of 266 archived horse serum samples collected from adult horses between January and March in 2012, 2015, 2019, 2021, and 2022 (Fig. 1, see Additional file 1, Table S2 for detailed information) were acquired from Finnish Food Authority. Most horses had been sampled for breeding purposes (182/266) or for diagnosing a disease (48/266). Additionally, 11 had been sampled for export and 25 for import reasons. Nine out of 48 horses that were sampled for disease diagnostics were suspected of having a respiratory infection, four had suspected equine viral arteritis and one a spontaneous abortion. The suspected disease was unknown for the rest (34) of these horses. Sample set included both racehorses and riding horses.

### Virus isolation attempt

Earlier, unsuccessful EqPPV isolation attempts have been done in baby hamster kidney cells, African green monkey kidney cells, and primary bovine oesophagus cells [3, 6, 9]. Here, equine dermis cells (NBL-6) and aneuploid human keratinocyte cells (HaCat, ATCC) were grown in DMEM High Glucose media (Sigma-Aldrich) supplemented with 10% (for maintenance) or 2% (for infection) heat inactivated fetal bovine serum (Gibco), 100 IU/ml of penicillin (Sigma-Aldrich), 100 µg/ml of streptomycin (Sigma-Aldrich), and 2 mM of L-glutamine (Sigma-Aldrich). HaCat and NBL-6 cells were seeded to 6-well dishes in density 100,000 cells/well day before the infection. Before inoculating the cells, dry swabs taken from skin lesions of horses suffering from pastern dermatitis [9] were incubated in 1 ml of Dulbecco’s phosphate buffered saline containing 0.2% of bovine serum albumin (DPBS + BSA) overnight at 4 °C. Samples were filtered with 0.45 µM polyethersulfone membrane filters and inoculated into the cells for 1 h at 37 °C, 5% CO_2_. Fresh culture medium was added. Cells were observed daily for cytopathic effect (CPE) and supernatants from each five passages were tested with EqPPV-specific probe-based PCR described in our earlier study [9]. DNA was extracted with Nucleospin tissue kit (Macherey–Nagel) according to manufacturer’s instructions.

### Preparing the antigen and immunofluorescence assay slides

An immunofluorescence assay (IFA) was set up with a recombinant protein technique based on B2L and F1L sequences from the first detected equine case (GenBank no. OQ110635.1, OQ248663.1) [3]. The B2L and F1L genes with 6 × Histidine (His) and Hemagglutinin (HA) tags on C-terminus were amplified by PCR from synthetic genes (Thermo Fisher Scientific), using appropriate primers with incorporated initiation and termination codons and flanked by KpnI and SgsI sites. Phusion High-Fidelity DNA polymerase (Thermo Fisher Scientific) was used for PCR amplification according to manufacturer’s instructions. The amplified fragments were digested with KpnI/SgsI restriction enzymes (Thermo Fisher Scientific) and cloned into the pCAGGS expression vector [19]. The constructed plasmids pCAGGS-B2L and pCAGGS-F1L were transfected into Vero E6 cells (ATCC) in a 100-mm plate using FuGENE HD transection reagent (Promega). The transfected cells were grown in Minimum Essential Medium Eagle (Sigma-Aldrich) supplemented with 10% fetal bovine serum (Gibco), 100 IU/ml of penicillin (Sigma-Aldrich), 100 µg/ml of streptomycin (Sigma-Aldrich), and 2 mM of L-glutamine (Sigma-Aldrich). Seventy-two hours post-transfection, the cells were harvested, pelleted at 4000 × g for 5 min at 4 °C, and washed twice with 1 × PBS. The washed transfected cells were placed on microscope slides, fixed with cold acetone for 10 min, and stored at −20 °C until use. The washed pelleted cells were also used as the antigen in immunoblotting.

### Immunofluorescence assay

All the serum samples were screened for IgG antibodies against both antigens, F1L and B2L. First, the slides were blocked with DPBS + BSA (25 µl/well) for 30 min in a 37 °C moist chamber and washed 3 × 5 min with PBS and once with ultrapure water (Milli-Q). Next, they were incubated in 37 °C moist chamber for another 30 min with serum samples diluted 1:20 in DPBS + BSA (25 µl/well). First, anti-HA antibodies (BioLegend) diluted 1:1000 and later, positive serum sample from a PCR-verified patient horse were used as positive controls. After washing the slides another 3 × 5 min with PBS and 1 × with water, they were incubated in 37 °C moist chamber for additional 30 min with 25 µl/well of Fluorescein (FITC)-conjugated AffiniPure Goat Anti-Horse IgG (H + L) (horse samples, Jackson ImmunoResearch; diluted 1:100 or 1:150 in DPBS depending on slide batch) or Anti-Mouse IgG (H + L) (HA control, diluted 1:100). Finally, slides were washed 3 × 5 min with PBS and once with water, dried, covered with mounting media (Epredia Immu-Mount, Thermo Scientific) and a cover glass, and checked with a fluorescent microscope (Zeiss Axioplan 2). All the IFA results were classified as positive, unclear, or negative. Clinical samples that were positive with both antigens were also titrated with a two-fold dilution series (1:20–1:320) against the B2L antigen.

Samples from horses with dermatitis and other samples from 2022 were further tested for IgM antibodies with B2L antigen. For IgM IFA, IgG was first removed with GullSORB IgG Inactivation Reagent (Thermo Scientific): one drop was mixed with 5 µl of serum, incubated for 10 min at room temperature (RT), centrifuged for 8 s in 11,000 × g, and diluted 1:2 to acquire 1:20 dilution. The IgM IFA was performed similarly to the IgG IFA except for that instead of 30 min, serum samples were incubated for 2.5 h, and FITC-conjugated Goat anti-Equine IgM Heavy Chain secondary antibody (Novus) with a 1:100 dilution was used as secondary antibody.

Samples showing high background fluorescence in IFA were repeated by first preadsorbing the sera with cells used in the transfection. Nontransfected Vero E6 cells were detached from confluent T75-bottles, washed with DPBS + BSA and split into 24 tubes. Cells were then fixed with 50 µl of + 4 °C acetone for 7 min after which the acetone was removed by centrifugation and pellets were airdried. Before the IFA, serum dilutions were mixed with the cell pellets and incubated 5–10 min at RT. Cells were pelleted again by centrifugation and the sera were used in IFA as described above. All the centrifugations were done at 500 × g for 5 min.

### Western blot

The antigens used in the IFA protocols were further assessed by testing three IFA-IgG-positive and one negative serum with Western blot (WB). First, Vero E6 cell pellets containing B2L and F1L antigens and pelleted antigen-free cells were suspended in 4 × Laemmli buffer and denatured for 5 min at 95 °C. Five µl of cell suspension was run in SDS-PAGE gel (Mini-PROTEAN TGX Stain-Free Gels, Bio-Rad) along with 3 µl of PrecisionPlus dual color protein standard (Bio-Rad) and transferred into Amersham Prothan 0.45 µm NC nitrocellulose blotting membrane (Cytiva). After blocking the membrane in 4% milk in tris-buffered saline (TBS, Medicago) overnight at 4 °C, membranes were incubated with diluted serum samples for 1 h at RT with shaking (PSU-10i Orbital Shaker, Biosan, 100 rpm). One of the positive samples was tested with 1:100 and 1:200 serum dilutions to estimate the correct dilution and the rest only with 1:200 dilution. Membrane was then washed 3 × 5 min in TBS + 0.05% Tween20 (TBST) and incubated with peroxidase-conjugated AffiniPure Goat Anti-Horse IgG (H + L) secondary antibody (Jackson ImmunoResearch) diluted 1:40,000 for another 1 h after which it was washed 3 × 5 min in TBST and 1 × 5 min in ultrapure water. The membrane was visualized with Pierce ECL Western Blotting Substrate (Thermo Scientific) according to manufacturer’s instructions and imaged on X-ray film (Fujifilm Super RX Medical X-ray film).

### Data analysis

For calculation purposes and to avoid false positive results, animals were considered seropositive for EqPPV if they showed a positive reaction with both antigens and unclear if they were positive with one antigen only. Samples that were negative with both antigens were considered seronegative. Similarity of seroprevalence between groups was tested with Fisher exact test with a significance level of 0.05. All the statistical tests were done with IBM SPSS Statistics 29.0.2.0. Pictures were drawn in Jupyter Notebook utilizing Pandas, SciPy, Matplotlib, Seaborn, and NumPy packages [20–24]. Confidence intervals were calculated with Wilson score method.

## Results

### Virus isolation

We attempted to cultivate five samples positive for EqPPV DNA in equine dermis cells (NBL-6) and aneuploid human keratinocyte cells (HaCat). CPE, characterized by rounded cells, was observed with both cell lines in 1 of the samples 7 days after infection (passage 1). However, based on a PCR performed on culturing media (Additional file 1, Table S3), CPE could not be confirmed to have been caused by EqPPV. The CPE was not seen in any further passages and Ct-values increased during each passage.

### Expression of EqPPV recombinant proteins and evaluating an immunofluorescence assay

**Fig. 2:**
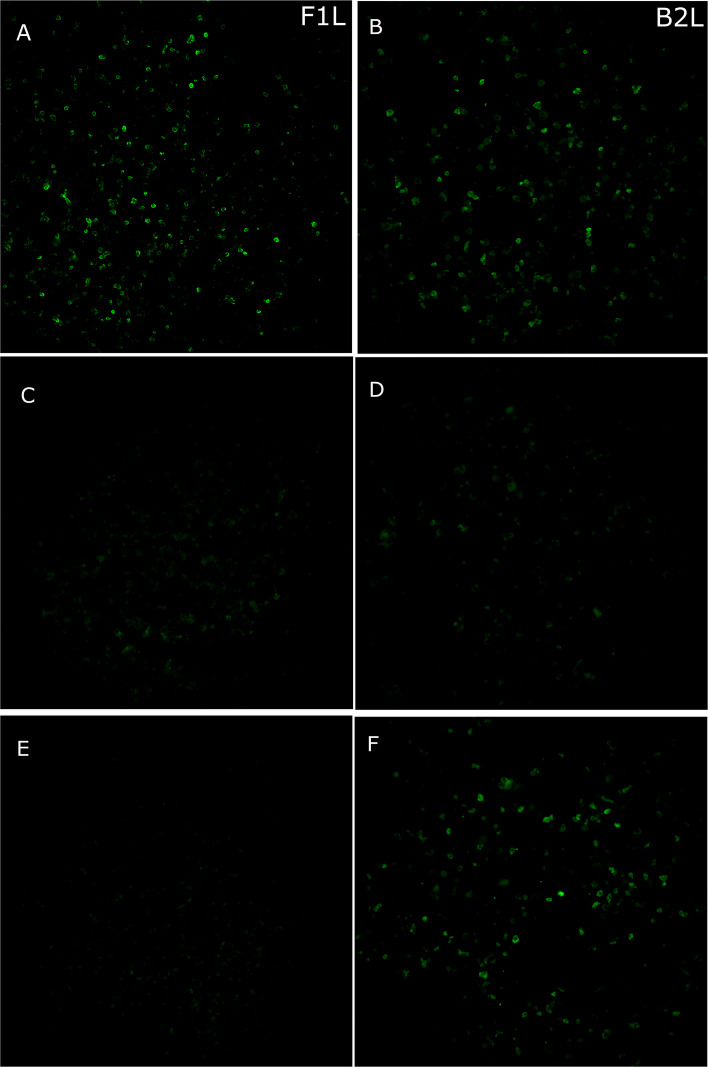
Equine parapoxvirus immunofluorescence assay. Positive serum samples (**A, B**), negative serum samples (**C, D**) and HA-control (**E, F**) with F1L (**A, C,** and **E**) and B2L (**B, D,** and **F**) antigens (× 20)

As the culturing attempts of EqPPV were repeatedly unsuccessful, and with almost full-length EqPPV genome sequence available [3], we concentrated our efforts on establishing serological assays based on antigenic recombinant proteins. As previous literature showed antigenicity of homologues B2L and F1L proteins of other PPVs, we selected these for further studies. Both proteins were successfully expressed with HA and His tags from the related ORFs from pCAGGS plasmids transfected in Vero E6 cells. In IFA, a control anti-HA antibody detected the C-terminal HA tag in B2L but not in F1L protein construct. This suggests that this epitope may be folded so that it is inaccessible to anti-HA antibodies (Fig. 2 and Additional file 2, Fig. S1). However, positive serum samples were identified with both antigens (detailed in the following sections). The four negative control horses sampled before the epidemic were negative, including the OPV-positive horse.

**Fig. 3:**
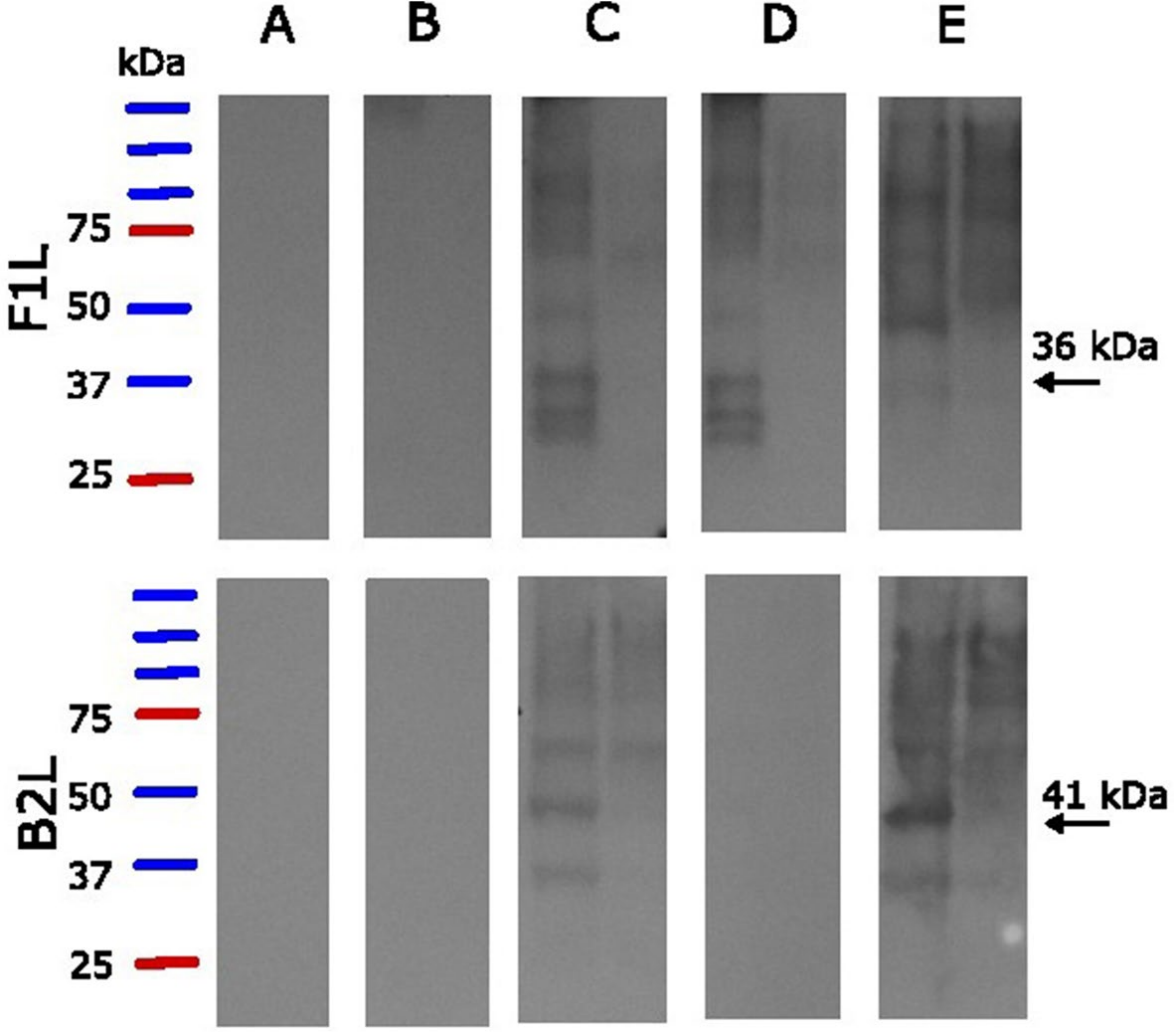
Equine parapoxvirus Western blot assay based on F1L and B2L antigens, used with horse sera. Serum from a EqPPV-PCR and IFA-negative horse (A), sera from PCR and IFA-positive horses collected < 1 week (B, horse B1) and 1–2 months (C-D, horse K1) after the onset of the disease, and serum from a horse that had a PCR and IFA-confirmed EqPPV infection 1 year earlier (E, horse C3). 1 st lane of each subpicture has cell suspension with the antigen, and the 2nd lane has cell suspension without the antigen. Sample from horse K1 was tested with two serum dilutions (C: 1:100 and D: 1:200). Other samples were tested with 1:200 dilution only. Expected protein sizes (36 kDa for F1L and 41 kDa for B2L) are marked with arrows. Each subplot represents an individual sample on a separate membrane and size markers (Precision Plus Protein Dual Color Standard) were included in each membrane (not shown separately for each subplot)

Based on the amino acid sequences, sizes of the proteins are 41 kDa (B2L) and 36 kDa (F1L)[9]. Bands of these sizes were detected in WB when using two IFA-positive serum samples against cell suspension containing these antigens whereas bands of these sizes were not detected when testing these sera against cell suspension without the antigens (Fig. 3 and Additional file 2, Fig. S2). No bands were detected with an IFA-negative control serum and in one IFA-positive sample of the three tested. Due to the novelty of the virus and the lack of a golden standard test, specificity and sensitivity could not be calculated.

All samples combined, IgG positivity and negativity to B2L and F1L were in 97.6% agreement in IFA. Only seven samples out of 294 gave conflicting results. In all the seven cases, antibodies to F1L were positive and to B2L either negative or unclear. These seven samples were considered unclear in the result analysis.

### Antibody response of horses with pastern dermatitis

In total, 79% (22/28) of samples collected from horses with pastern dermatitis between December 2021 and March 2022 were IgG positive with B2L, 86% (24/28) with FL1, and 79% (22/28) with both (Table 1). Seventy-three percent (73%; 11/15) of the horses sampled within a week from onset and 82% (9/11) sampled between a few weeks and few months were positive with both B2L and F1L. The two horses (C3 and I1) that were resampled a year after a PCR-verified infection were still IgG positive in both assays based on limited follow-up. Sera from two horses only were negative with both antigens, D2 [sampled 1-day post-onset, borderline case in PCR] and F4 [sampled 4 days post-onset, positive in PCR] (Additional file 1, Table S1). Serum from one horse, F1, [sampled between a few days and a few weeks, positive in PCR]) provided unclear result with both antigens. The results of sera from three horses (F3, G1 and H1) differed between antigens: all were positive with F1L but negative or unclear with B2L. The only sample that was negative in PCR [9], J1, [sampled within a few days post-onset] was IgG positive with both antigens. Geometric mean titer of the positive samples was 39.8 (titer range from 20 to 160, Table S1) and no variation was detected between sampling groups.

**Table 1:** The proportion of IgG positive cases among horses with pastern dermatitis, December 2021—March 2022

Sampling time after first detection of signs	Seroprevalence (B2L)^a^, %	Seroprevalence (F1L)^a^, %	Seroprevalence (Both)^a^,%
A few days	73.3 (11/15)	86.6 (13/15)	73.3 (11/15)
A few days-few weeks	81.8 (9/11)	90.9 (10/11)	81.8 (9/11)
1 year^b^	100.0 (2/2)	100.0 (2/2)	100.0 (2/2)
Total	78.6 (22/28)	85.7 (24/28)	78.6 (22/28)

^a^Reported as % (n/N)

^b^Both horses were also sampled earlier

No clear positive samples were identified with IgM IFA. Four samples gave unclear results that could not be clearly classified as positive or negative. Three of these were from horses sampled within a week from the first detection of the clinical signs and one was sampled over 3 weeks after the onset.

### EqPPV seroprevalence in Finland between 2012–2022

**Fig. 4:**
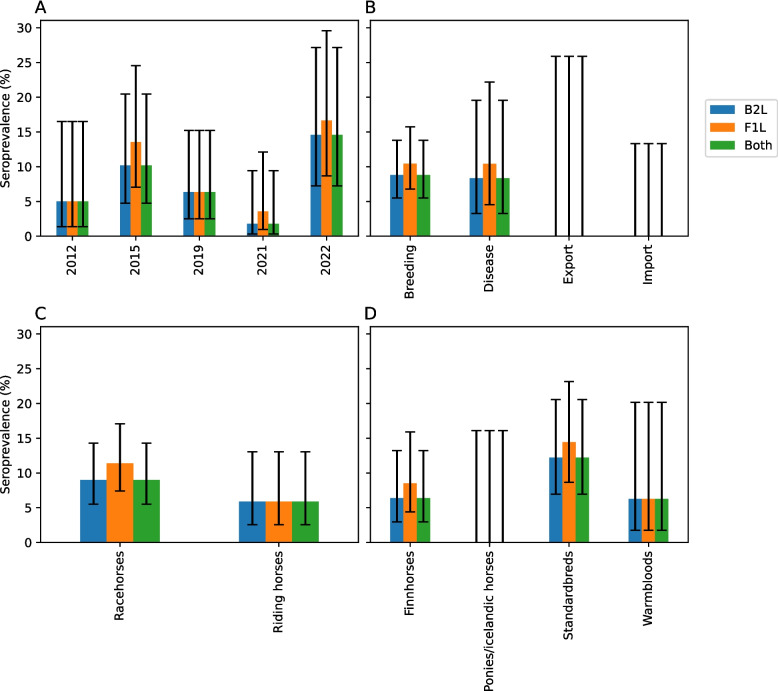
Equine parapoxvirus IgG seroprevalences in Finnish horses. Samples have been classified by collection year (**A**), the reason for collection (**B**), use of the horse (**C**), and horse type (**D**). Error bars represent 95% confidence intervals (Wilson score method)

Seroprevalence of horses to EqPPV in Finland varied between 1.8–14.6% (B2L, p = 0.101) and 3.6 and 16.7% (F1L, p = 0.096, Table S4, Additional file 1) between five time-points. Highest seroprevalences were detected in 2015 and 2022 with both antigens and the lowest in 2021 (Table 2 and Fig. 4). Seroprevalence was highest in horses sampled for breeding or disease diagnostics. None of the exported or imported horses were seropositive. No significant differences were detected between racehorses and riding horses (B2L, p = 0.224; F1L, p = 0.073). No antibodies were detected in ponies, whereas total seroprevalence in horses was 8.5% (B2L, p = 0.382; F1L, p = 0.232).

**Table 2:** Seroprevalences and Wilson score intervals (95% confidence level) grouped by sampling year, use of the horse, reason for sampling, and horse type. Numbers of positive samples and total samples are reported in brackets

Class	B2L		F1L		Both	
	Prevalence (%)	95% CI	Prevalence (%)	95% CI	Prevalence (%)	95% CI
2012	5.0 (2/40)	1.4–16.5	5.0 (2/40)	1.4–16.5	5.0 (2/40)	1.4–16.5
2015	10.2 (6/59)	4.7–20.5	13.6 (8/59)	7.0–24.5	9.8 (6/61)	4.6–19.9
2019	6.4 (4/63)	2.5–15.2	6.4 (4/63)	2.5–15.2	6.4 (4/63)	2.5–15.2
2021	1.8 (1/56)	0.3–9.5	3.6 (2/56)	1.0–12.1	1.8 (1/56)	0.3–9.5
2022	14.6 (7/48)	7.3–27.2	16.7 (8/48)	8.7–29.6	14.6 (7/48)	7.3–27.2
Racehorses	9.0 (15/167)	5.5–14.3	11.4 (19/167)	7.4–17.1	9.0 (15/167)	5.5–14.3
Riding horses	5.9 (5/85)	2.5–13.0	5.9 (5/85)	2.5–13.0	5.9 (5/85)	2.5–13.0
Others	0.0 (0/2)	0.0–65.8	0.0 (0/2)	0.0–65.8	0.0 (0/2)	0.0–65.8
Breeding	8.9 (16/182)	5.5–13.8	10.4 (19/182)	6.8–15.7	8.9 (16/182)	5.5–13.8
Import	0.0 (0/25)	0.0–13.3	0.0 (0/25)	0.0–13.3	0.0 (0/25)	0.0–13.3
Export	0.0 (0/11)	0.0–25.9	0.0 (0/11)	0.0–25.9	0.0 (0/11)	0.0–25.9
Disease	8.3 (4/48)	3.3–19.6	10.4 (5/48)	4.5–22.2	8.3 (4/48)	3.3–19.6
Finnhorses	6.4 (6/94)	3.0–13.2	8.5 (8/94)	4.4–15.9	6.4 (6/94)	3.0–13.2
Standardbreds	12.2 (11/90)	7.0–20.6	14.4 (13/90)	8.6–23.2	12.2 (11/90)	7.0–20.6
Warmbloods	6.3 (2/32)	1.7–20.1	6.3 (2/32)	1.7–20.1	6.3 (2/32)	1.7–20.1
Ponies/Icelandic horses	0.0 (0/20)	0.0–16.1	0.0 (0/20)	0.00–16.1	0.0 (0/20)	0.0–16.1
Others	0.0 (0/2)	0.0–65.8	0.0 (0/2)	0.00–65.8	0.0 (0/2)	0.0–65.8

**Fig. 5:**
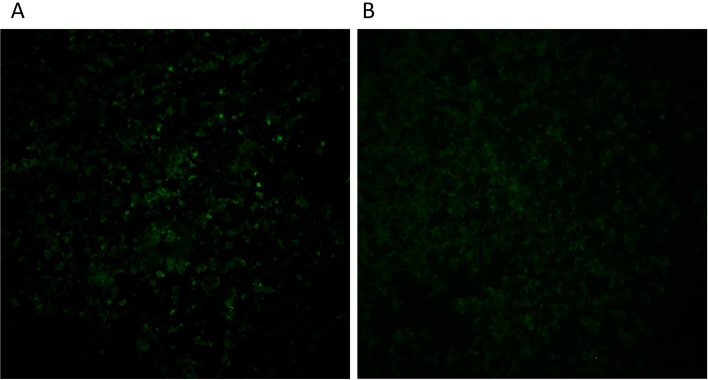
IgM IFA. A possible IgM positive serum sample (**A**) and IFA negative sample (**B**) from early 2022 (× 20)

Out of the positive horses, one lived in Uusimaa region (Southern Finland), five in Ostrobothnia (Western Finland), and one in Savonia (Eastern Finland, location unknown for the rest of the horses). Three of the positive horses had been brought to Finland from North America (#71, #94, and #137) and one from Germany (#211). Due to the frequent travelling of many of the horses, no detailed geographical analysis was done. One of the horses (#237) sampled in 2022 was positive for IgM (Fig. 5, Table S2 in Additional file 1 and Fig. S3 in Additional file 2) and one (#231) could not be clearly defined as positive or negative. Both horses were sampled for breeding purposes in February.

## Discussion

Here, we developed a serological method to detect antibodies raised by a novel parapoxvirus, EqPPV, which caused a widespread pastern dermatitis epidemic among horses in Finland during winter 2021–2022. With this method, we analyzed serum samples from affected horses as well as archived samples to acquire information on immune response, prevalence, and history of EqPPV.

Serological data demonstrate that the infection stimulates a quick immune response as IgG antibodies could be detected in most affected horses during the first few days after onset. This is faster than expected based on knowledge on other PPVs [25, 26]. For example, IgG response was observed around two weeks after the primary infection in experimental infections of sheep with ORFV [26] and cows with BPSV [25]. Some of the difference could be explained by the fact that the IgG response in experimental infections has been measured from the infection whereas we measured it from the onset of clinical signs. However, since reports from the field suggest that the incubation time likely is no more than a few days, IgG response can still be considered fast [9]. Due to the small seroprevalence in general population in 2021, it is unlikely that antibodies from previous infections act as a confounding factor.

Two horses were sampled a year after the disease, and both were still seropositive. Despite other PPVs generally causing only a short-lived protective immunity [26–28], a detectable antibody response has been observed in a cow experimentally infected with BPSV even three years after the infection [25]. In association with EqPPV infection, a follow-up longer than one year with a larger sample size would be needed to determine the duration of IgG response.

IFA was able to detect IgG with both the antigens with good agreement (only 7/249 samples giving conflicting results). Detection by HA antibody failed with F1L, possibly due to the short length of the HA tag which may be folded so that is inaccessible for the antibodies. The WB analysis confirmed the existence of proteins that were the expected sizes and were not detectable in antigen-free cell suspension in two out of three tested positive samples. Bands that were detected in antigen-free suspension as well are likely reaction with some other component of the cells. The negative result of the third IFA positive sample in WB can potentially be explained by a higher serum dilution: when one of the IFA positive samples was tested with two different serum dilutions (1:100 and 1:200), the result was positive with 1:100 dilution only. In literature, performance of IFA in relation to WB varies depending on the application. They have been shown to have a good agreement e.g. with Human Immunodeficiency Virus [29] whereas the specificity of WB was better than that of IFA when separating different hantaviruses [30].

As this is the 1 st serological assay set up for this virus, specificity and sensitivity of the IFA could not be calculated. Cross-reaction with other poxviruses that can infect horses (e.g. OPVs and molluscum contagiosum-like virus [31]) cannot be excluded. However, considering that the OPV-positive serum sample was negative, cross-reaction is unlikely. Cross-reaction with other PPVs remains possible, but those have not been reported in horses. To maximize sensitivity, F1L antigen should be used in screening, because a few samples were positive only with this one. This implies that F1L is either slightly more sensitive or less specific than B2L. Our approach—necessitating positivity against both F1L and B2L for a sample to be considered positive—was strict, as we wanted to avoid potential overestimation of the prevalence. This may, however, underestimate the seroprevalence.

No clear IgM antibodies were detected in horses that had pastern dermatitis. However, possible positive samples were detected in archived samples from early 2022. Detecting IgM in general horse population at that time is possible as that was the peak of the epidemic. We don’t know if these horses were clinically healthy, nor if asymptomatic infections are possible. Still, the result should be considered uncertain as IgM can sometimes be cross-reactive to other sample components [32]. In clinical cases, the short half-life of IgM [33] may have led to it being below detection limit by the time the lesions were spotted and sampled. These IgM assays could also lack sensitivity, and the results should be considered uncertain due to the lack of a positive control for protocol optimization. In comparison, ORFV has been shown to induce a detectable IgM response in most animals showing clinical signs [34].

Data on archived samples indicate that EqPPV has been circulating in the Finnish horse population for several years. Based on one equine serum sample from 2012 that was IgG-antibody-positive with both antigens, the virus has been circulating even before the first report in 2013. Peaking seroprevalences were detected in 2015 and 2022, well in line with the field reports about epidemics. However, the differences between years were not statistically significant, possibly impacted by our limited sample size.

Regarding epidemiological aspects related to the serological findings, the ≥ 10 km distances between the stables, at which horses with pastern dermatitis were sampled during the epidemic 2021–2022, makes airborne transmission unlikely. All these horses were trotting racehorses (Additional file 1, Table S1). This is in line with the previously recognized pastern dermatitis risk factors: having racehorses or having attended a race event [9]. In this study, we found no further explanatory factor from either herd size or management system (Additional file 1, Table S1). Limited number of stables also prevents strong conclusions.

The 2021–2022 epidemic was only reported in racehorses. In archived samples, antibodies were also found in riding horses. It is important for riders to be aware that their horses can also be infected and competition events should be avoided if horses show signs of EqPPV infection. No antibodies were detected in ponies. This should, however, be interpreted with caution as the number of sampled ponies was small. It should also be noted that some sampling biases may be caused by different distribution of horse uses, types, and reasons for sampling between groups (Fig. 1).

To our knowledge, this is the 1 st serological study on this novel virus and provides new information for predicting and controlling future epidemics. Based on the reports from the field and the results of this study, further epidemics are likely within the next few years. No cases have been reported in horses outside Finland so far. Considering the findings in humans in the USA and racehorses travelling between countries, underdiagnosis is possible. In the future, serological screening should be broadened to other countries to understand the global distribution of the virus.

## Conclusions

This first serological study on EqPPV shows that the virus causes a fast immune response that can be detected with IFA for at least a year. It also shows that the virus has been circulating in Finland at least since 2012 and supports reports that EqPPV can cause reoccurring epidemics. In the future, screening of larger sample panels is needed to confirm connection between EqPPV and earlier epidemics as well as to understand the global distribution of the virus.

## Supplementary Information

Supplementary Material 1.

Supplementary Material 2.

## Data Availability

All data generated or analysed during this study are included in this published article and its Additional files.

## Abbreviations

ORFV: Orf virus
EqPPV: Equine parapoxvirus
BPSV: Bovine papular stomatitis virus
PCPV: Pseudocowpoxvirus
RDPV: Red deer parapoxvirus
GSEPV: Grey sealpox virus
OPV: Orthopoxvirus
CPE: Cytopathic effect
HA: Hemagglutinin
DPBS: Dulbecco’s phosphate buffered saline
IFA: Immunofluorescent assay
WB: Western blot
