# Supplementary material for: Antibody responses to equine parapoxvirus reveal a re-emerging pattern
Source: BMC Vet Res. 2026 Jan 24;22:111. doi: 10.1186/s12917-026-05314-0 (PMC12911042; doi:10.1186/s12917-026-05314-0)
Supplement: Supplementary file 2 — Supplementary Material 2. [file 12917_2026_5314_MOESM2_ESM.docx]

# Additional file 2: Raw versions of figures 2, 3, and 5

A


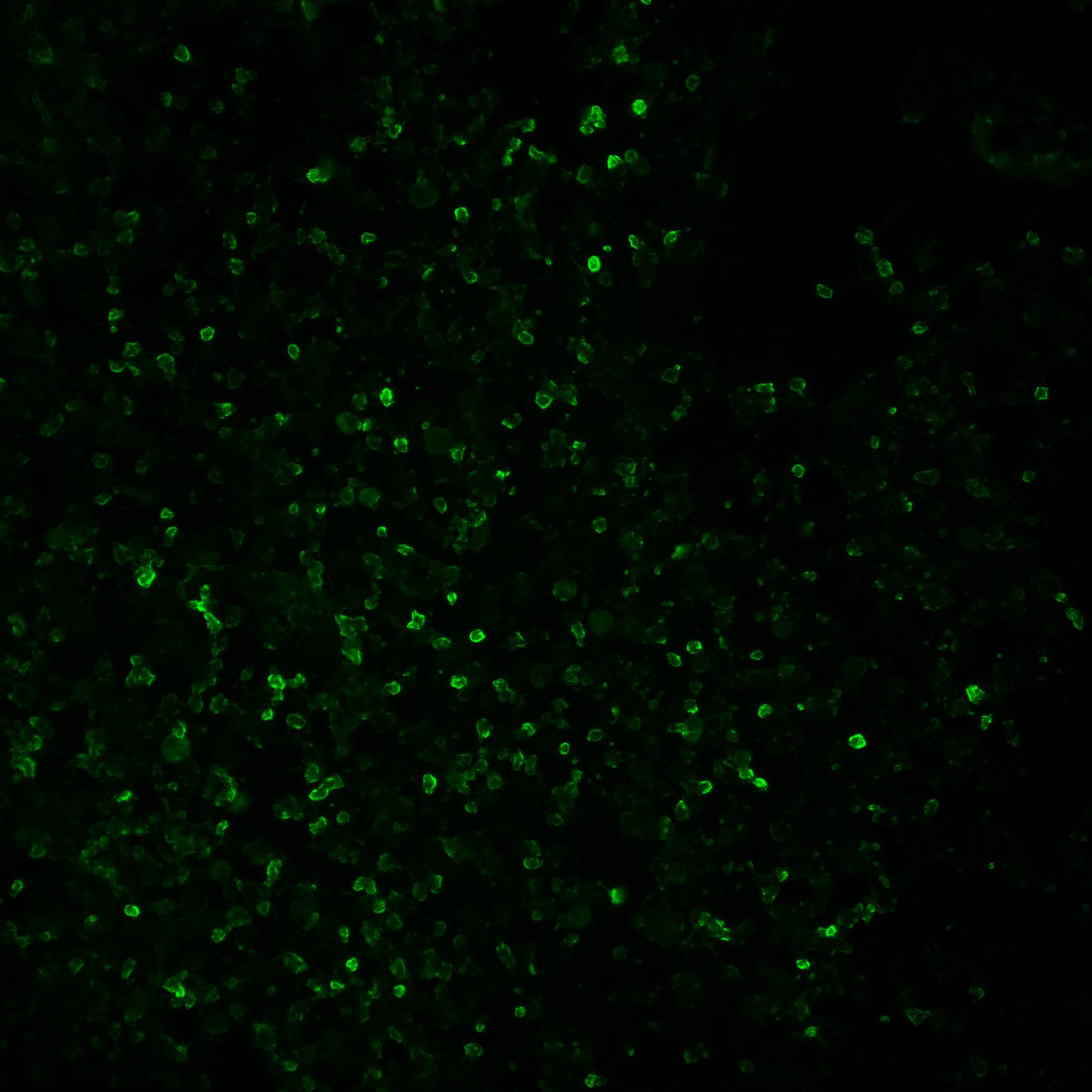


B


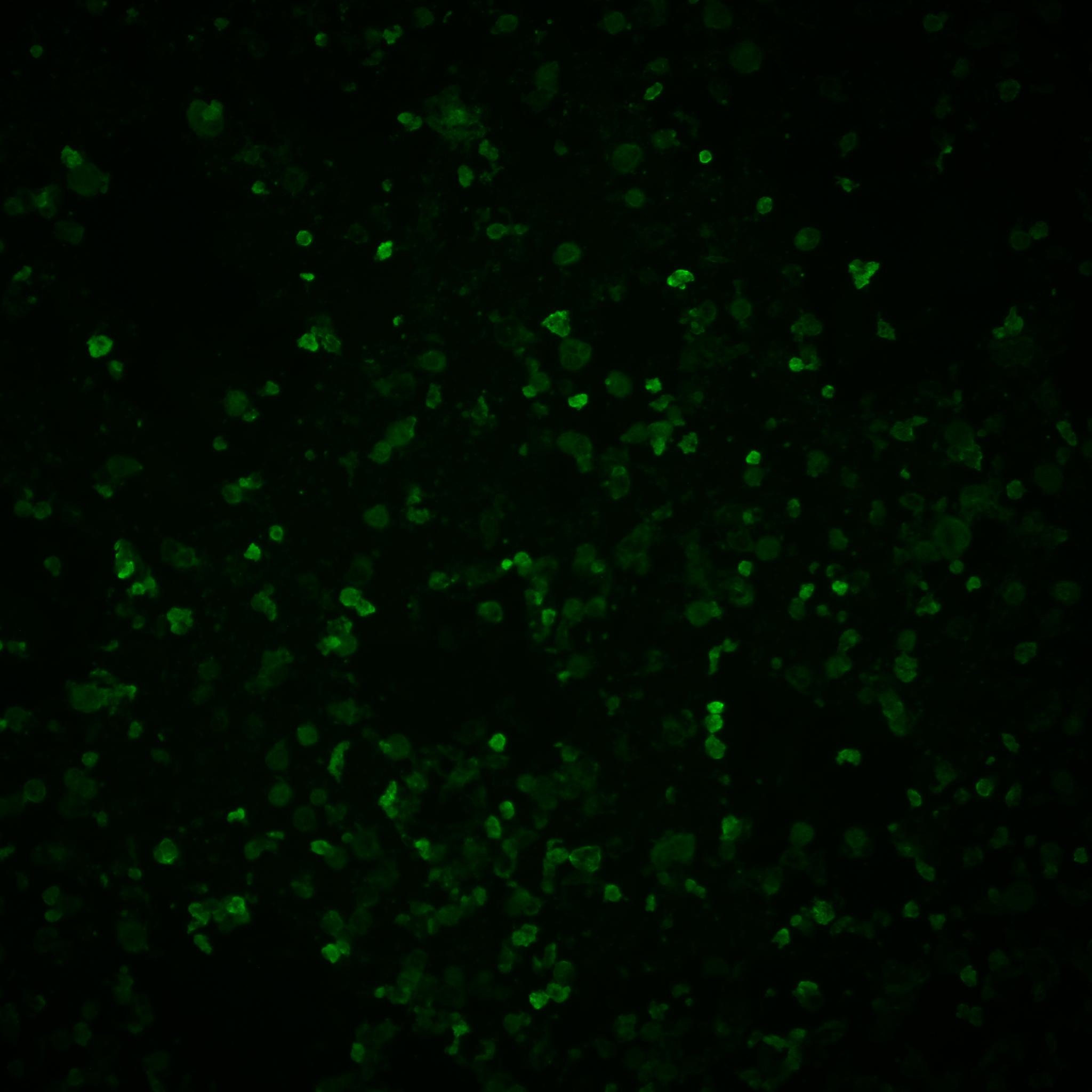


C


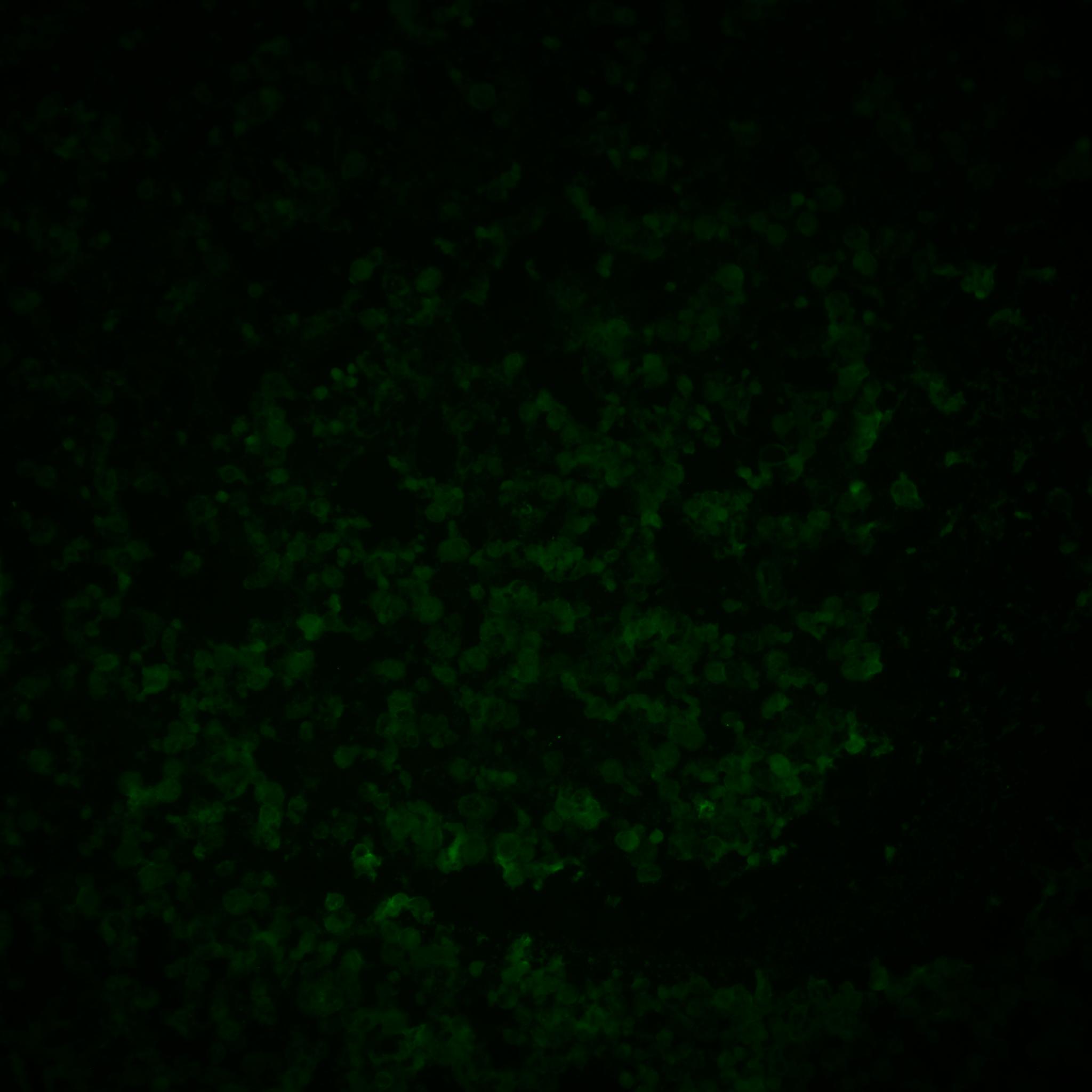


D


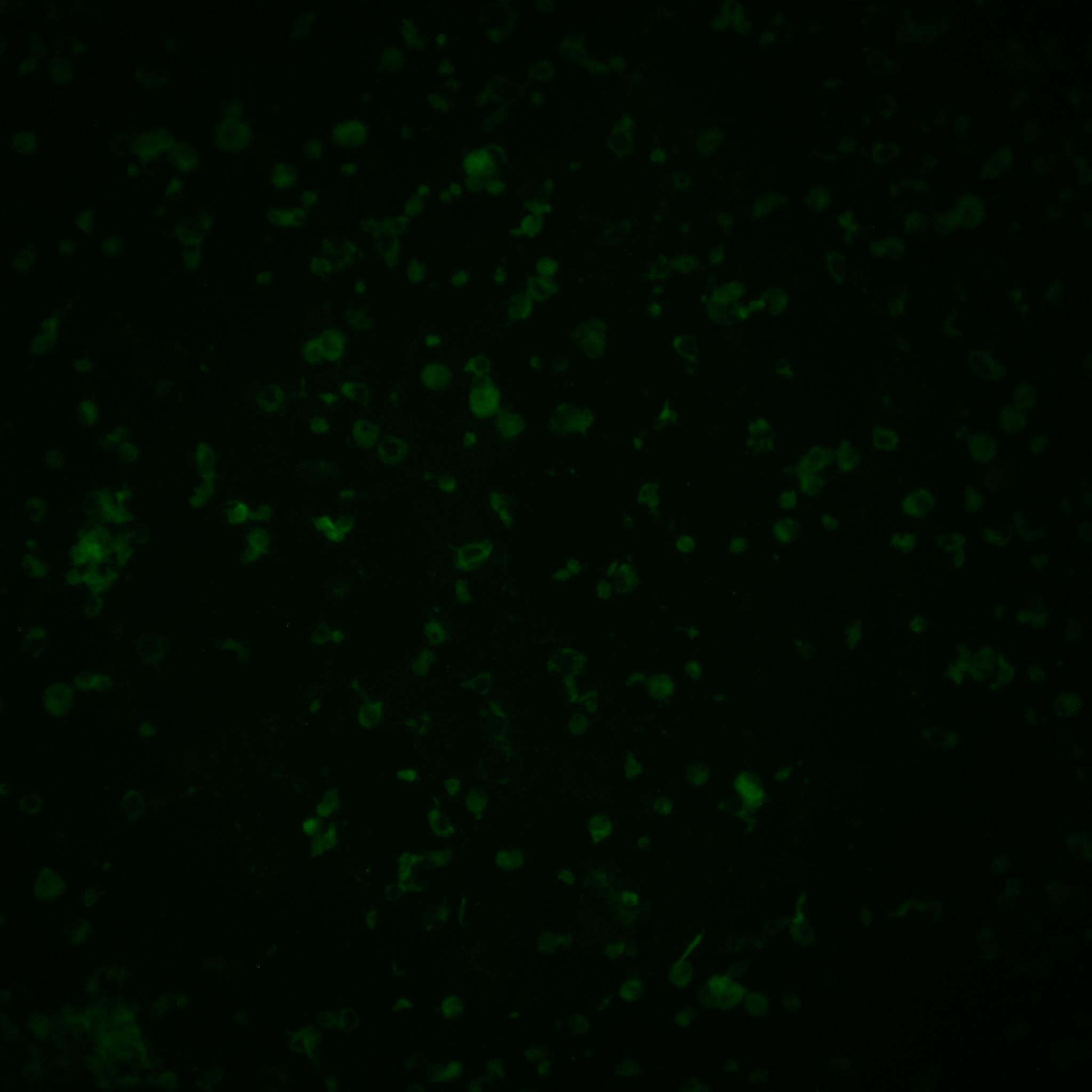


E


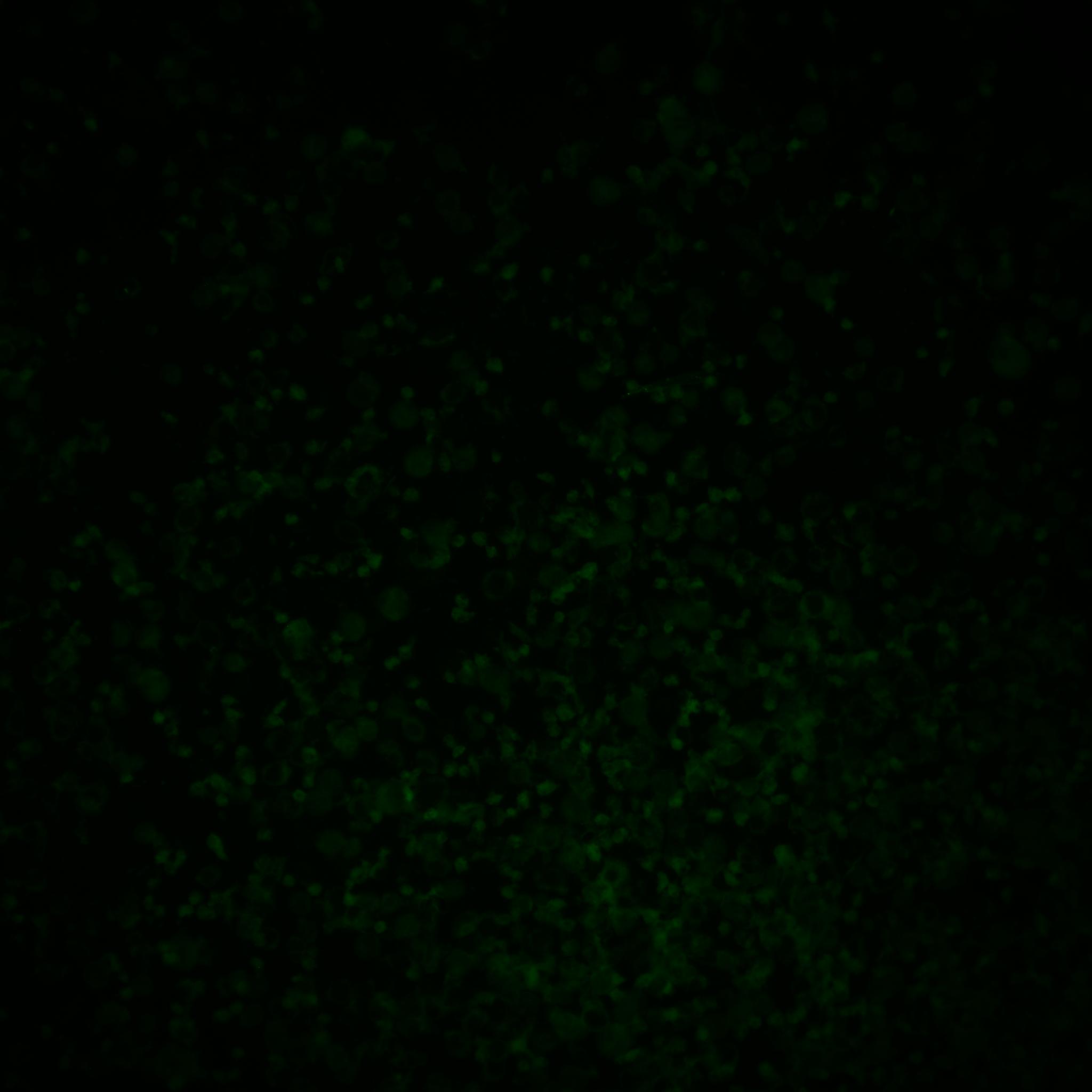


F


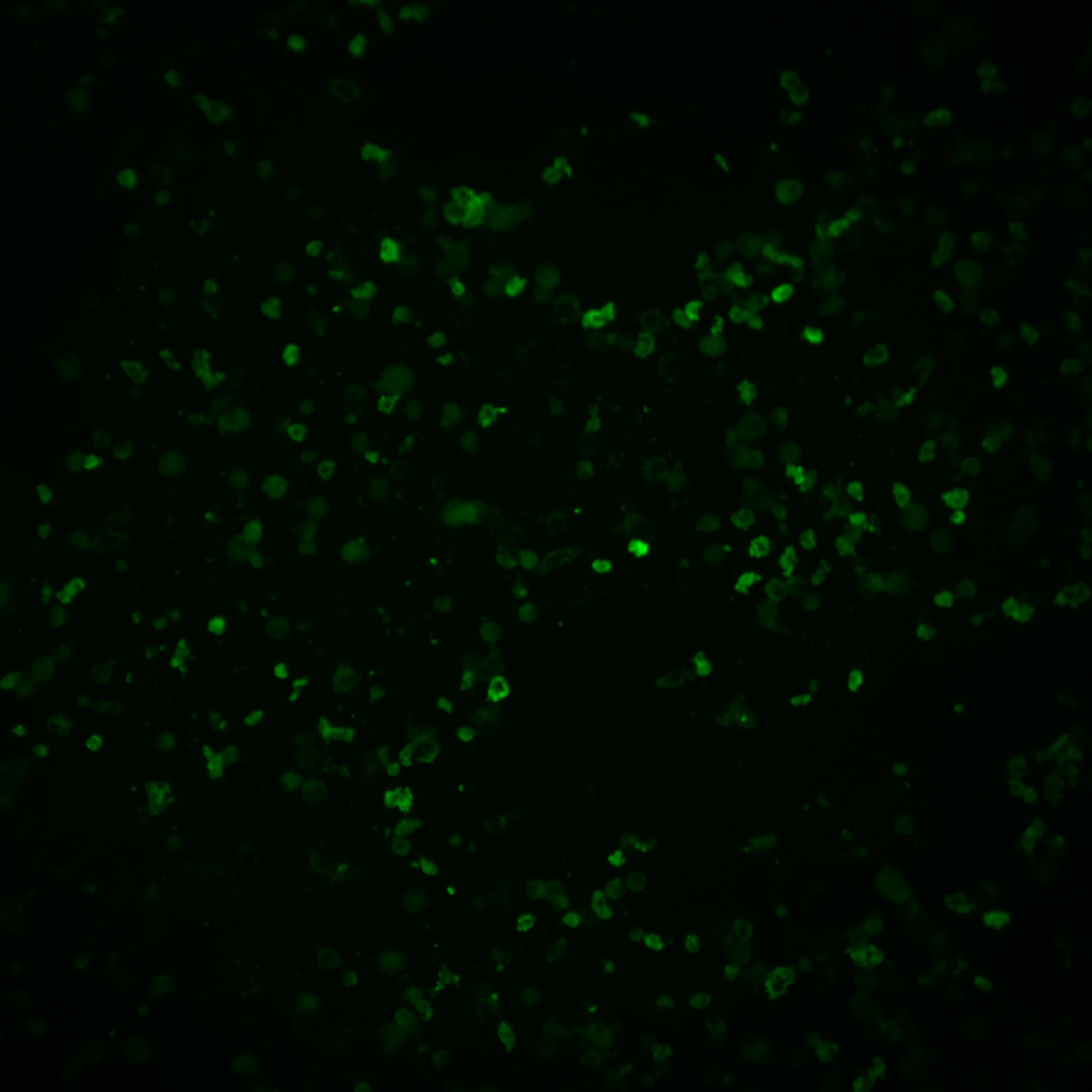


**Fig. S1.** Raw versions of Fig. 2. Positive serum samples (**A, B**), negative serum samples (**C, D**) and HA-control (**E, F**) with F1L (**A, C,** and **E**) and B2L (**B, D,** and **F**) antigens.


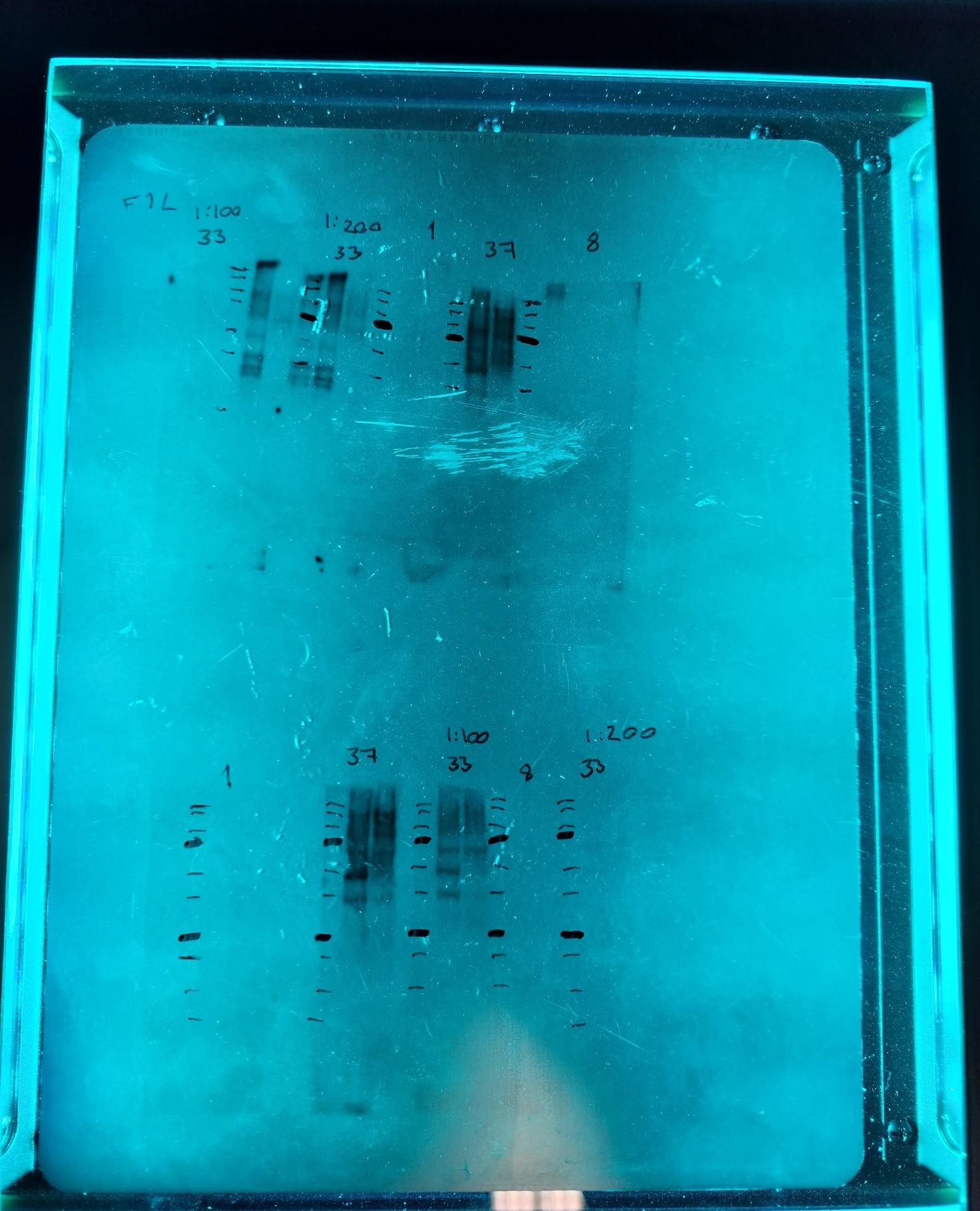


**Fig. S2.** Uncropped version of Fig. 3. Serum from a EqPPV-PCR and IFA-negative horse (1 [A in the manuscript]), sera from PCR and IFA-positive horses collected <1 week (8 [B in the manuscript], horse B1) and 1-2 months (33 [C-D in the manuscript], horse K1) after the onset of the disease, and serum from a horse that had a PCR and IFA-confirmed EqPPV infection 1 year earlier (37 [E in the manuscript], horse C3). Upper part represents F1L and lower part B2L. 1st lane of each subpicture has cell suspension with the antigen, and the 2nd lane has cell suspension without the antigen. Sample from horse K1 was tested with two serum dilutions (C: 1:100 and D: 1:200). Other samples were tested with 1:200 dilution only. Each subplot represents an individual sample on a separate membrane and size markers (Precision Plus Protein Dual Color Standard) were included in each membrane.

A


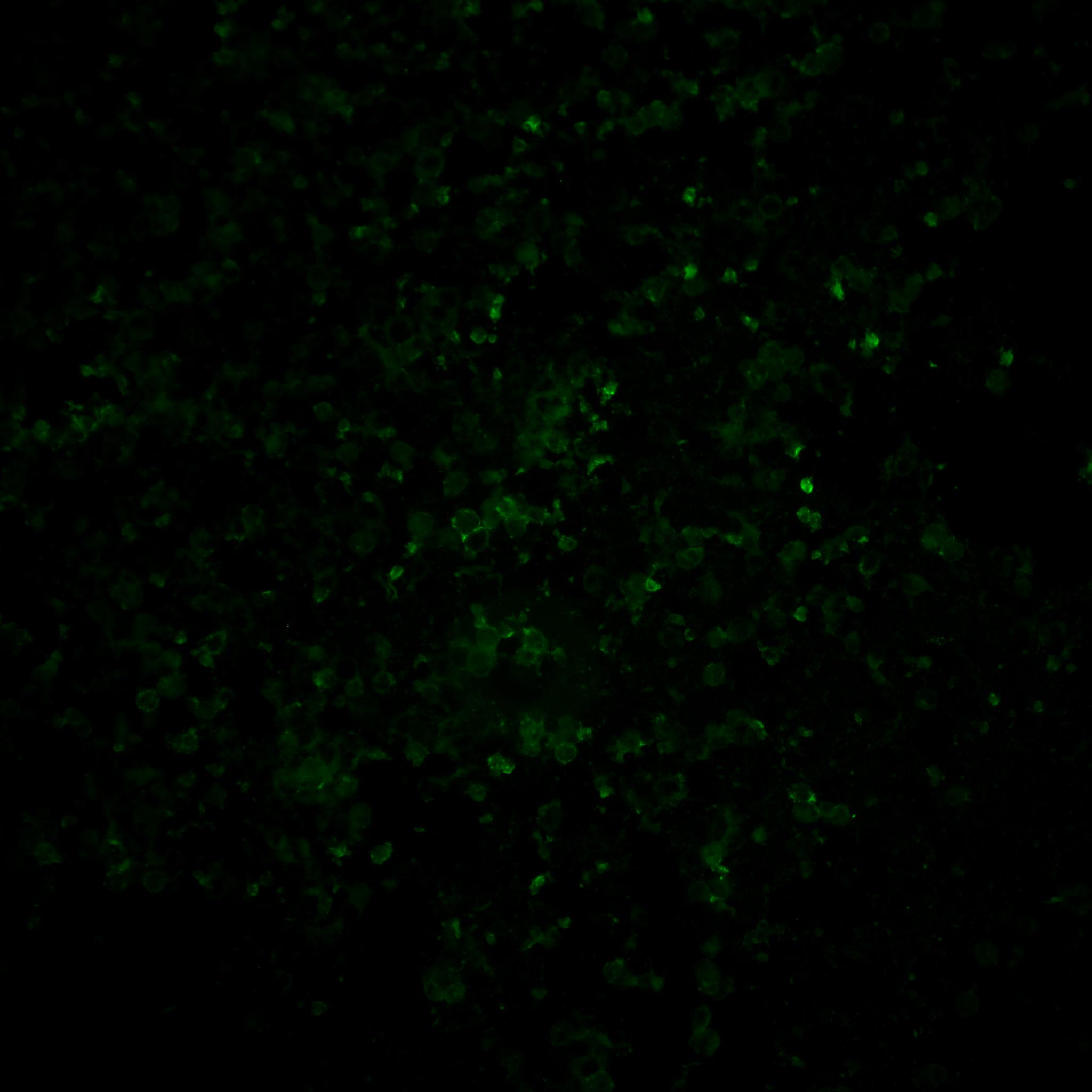


B


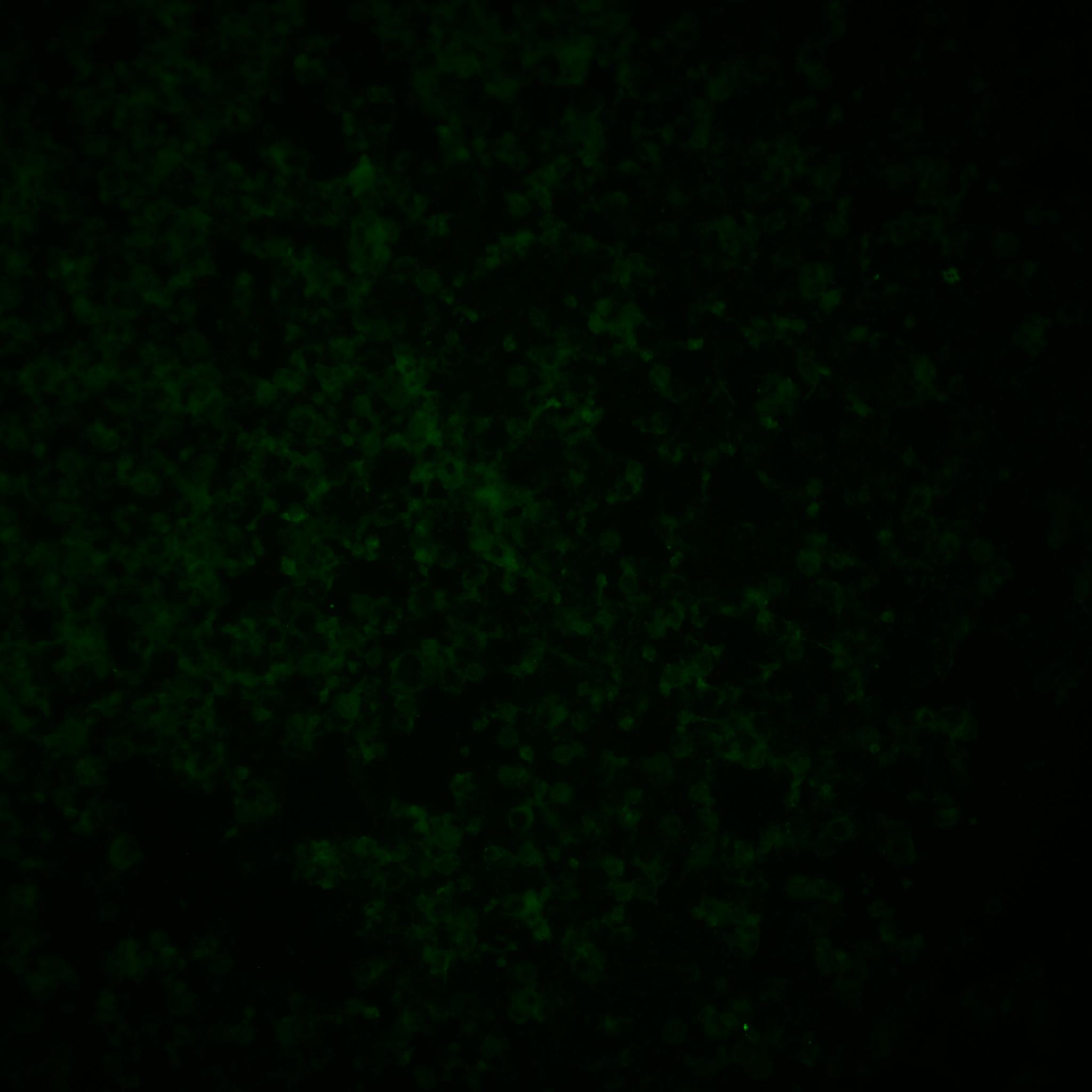


**Fig. S3.** Raw version of Fig. 5. A possible IgM positive serum sample (A) and IFA negative sample (B) from early 2022 (x20).
